# Integrated Genomic and Phenotypic Analyses Reveal Convergent Resistance Patterns in Clinical *Candida tropicalis* Isolates

**DOI:** 10.1111/myc.70181

**Published:** 2026-04-27

**Authors:** Christy Chedraoui, Nour Fattouh, Setrida El Hachem, Tsolaire Sourenian, Louna El Bitar, Lama El Moheb, Anna‐Souraya Sleiman, Ibrahim Bitar, Rola Husni, Roy A. Khalaf

**Affiliations:** ^1^ Department of Biological Sciences Lebanese American University Byblos Lebanon; ^2^ Department of Biology Saint George University of Beirut Beirut Lebanon; ^3^ Department of Microbiology, Faculty of Medicine University Hospital in Pilsen, Charles University Pilsen Czech Republic; ^4^ Biomedical Center, Faculty of Medicine Charles University Pilsen Czech Republic; ^5^ School of Medicine Lebanese American University Byblos Lebanon; ^6^ Lebanese American University Medical Center Rizk Hospital Beirut Lebanon

**Keywords:** adhesion, biofilm, *Candida tropicalis*, chitin, efflux‐pumps, ergosterol, resistance, virulence

## Abstract

**Background:**

The incidence of *Candida tropicalis* isolation is increasing in hospital settings. High azole resistance and mortality rates make it a pathogen that requires further analysis.

**Methods:**

Fourteen azole resistant *Candida glabrata* clinical isolates were collected from a Lebanese hospital and analysed through whole genome sequencing for single nucleotide polymorphisms in key resistance and virulence genes, and for phylogenetic relatedness. Isolates were then characterised for pathogenicity related attributes.

**Results:**

All isolates had Lys314Glu mutation in *ERG20* with multiple isolates displaying numerous shared mutations, such as Glu291Lys in *CDR2* and Ala16Thr in *CDR3*. With the exception of two isolates that clustered together, most isolates were over 99.6% identical based on a genomic heatmap, implying high relatedness consistent with localised clonal expansion, although SNP differences appeared too high to support this. However, the isolates exhibited increased ergosterol and chitin content, as well as upregulation of drug efflux pumps resulting in drug resistance.

**Conclusion:**

Our hospital isolates showed convergent resistant pathways, with many isolates having both shared and unique mutations and a high degree of genomic similarities.

## 1 Introduction

Candidiasis is an inflammatory, opportunistic infection caused by members of the *Candida* genus. These eukaryotic organisms inhabit various parts of the healthy human body, such as the oral cavity, gastrointestinal tract and urogenital areas. Such yeasts are often harmless but can become pathogenic under specific conditions. The rate of species incidence varies by infection site and geography [1, 2]. Candidiasis occurs frequently in immunocompromised patients and the elderly. It can be lethal when disseminated in the blood, leading to candidemia. *Candida albicans* has been the main cause of Candidiasis for decades, but the incidence of other non‐albicans species are continuously on the rise, including *Candida tropicalis* [3, 4, 5]. This is significant, as *C. tropicalis* candidemia has an overall reported mortality rate between 30% and 70%, which is generally higher than that of other *Candida* species [4, 6, 7]. Additionally, while the presences of *C. albicans* can occasionally be commensal, *C. tropicalis* is frequently correlated with fungal infections [8]. The fungus is mostly found in intensive care units (ICU), as well as in patients with catheters, malignancy and neutropenia [9]. Like *C. albicans* , *C. tropicalis* belongs to the CTG‐clade, is a diploid, dimorphic yeast with a parasexual mating cycle and has the ability to undergo phenotypic switching [10]. *C. tropicalis* can be seen as ellipsoidal budding cells or pseudomycellium, and, similar to *C. albicans* , can form true hyphae [11]. The latter mechanism acts as an important virulence factor that allows the yeast to adapt to different host environments and evade immune responses [7]. Compared to *C. albicans* and other *Candida* species, there are fewer studies examining the epidemiology and virulence factors of *C. tropicalis* , especially in a small country like Lebanon. One study characterising the susceptibility rate of non‐*albicans Candida* species from Lebanese hospitals found that *C. tropicalis* was the second most isolated species, accounting for 23.1% of all collected isolates [12]. Another study found *C. tropicalis* as the third most common non‐albicans *Candida* species in North Lebanon [13]. This exemplifies the need for further insight into the fungal pathogen. However, no study to date has addressed the molecular mechanisms of resistance and linked it to pathogenicity related phenotypic attributes.

*C. tropicalis* maintains its pathogenicity by a multitude of virulence factors. It can secrete numerous extracellular enzymes, produce strong biofilms and exhibit antifungal resistance [9, 11]. Currently, the most commonly used antifungals belong to the azole class; these target the *ERG11* gene product, 14α‐demethylase, to disrupt the fungal cell membrane [14, 15]. The rise in *C. tropicalis* infections correlates with an increase in azole drug resistance; the yeast species is less susceptible to the most common azole drug, fluconazole. One study found that the rate of resistance of *C. tropicalis* to fluconazole in China increased from 5.7% to 31.8% in less than ten years [16]. Other studies have also documented resistance to other antifungal drug classes, such as echinocandins, which target the *FKS1* and *FKS2* gene product, β‐1,3‐glucan synthase. These drugs target the fungal cell wall [17]. This rise in resistance is largely due to the widespread and liberal use of antifungal drugs, a phenomenon not limited to the yeast pathogen, as noted by the WHO [18, 19]. Species surveillance should be a major focus because the differences in *Candida* species may necessitate different treatments, despite their similarities.

The aim of this study is to characterise *C. tropicalis* isolates from a Lebanese tertiary care centre. The study involves determining resistance rates to numerous antifungal drugs and performing whole‐genome sequencing to examine the molecular mechanism of resistance, identify key mutations in resistance and pathogenicity related genes. In addition, single nucleotide polymorphisms (SNPs) analysis will be done to examine strain clonality and relatedness within the hospital. Phenotypic assays involving pathogenicity attributes such as virulence, adhesion and biofilm formation will also be performed.

## 2 Materials and Methods

### 2.1 *Candida tropicalis* Identification

Twenty‐five presumed *C. tropicalis* isolates were obtained from one Lebanese hospital and were analysed in a previous study. These isolates were obtained from a collection of 1000 clinical samples taken from urine, vaginal swabs, sputum, blood, cerebrospinal fluid and miscellaneous samples [12]. All isolates were stored in 30% glycerol cryogenic tubes. These isolates were stored in a deep freezer at −80°C. Isolates were first identified as *C. tropicalis* species by streaking on CHROMagar media and further confirmed through whole‐genome sequencing. Microbial identification and quality control were performed by MicrobesNG. Species identity of the isolates was determined using the taxonomic classification tool Kraken, which assigns sequencing reads to taxa based on k‐mer matches against a reference genome database. Ribosomal RNA sequences, including the 18S rRNA and small subunit (SSU) regions, were also extracted and compared against reference databases.

### 2.2 Antifungal MIC Determination

Minimum inhibitory concentrations were determined by broth microdilution after 24 and 72 h of incubation according to the CLSI M27 and M60 documents (Reference Method for Broth Dilution Antifungal Susceptibility Testing of Yeasts; Approved Standard—Second Edition—CLSI) and re‐analysed according to CLSI M60 ‘Performance Standards for Antifungal Susceptibility Testing of Yeasts’ after the second version of this document was issued (2020). Each sample was run in duplicate to ensure accuracy of the results. The drugs tested for were in the categories of azoles (Fluconazole, Posaconazole and Voriconazole) and echinocandins (Caspofungin, Micafungin and Anidulafungin) [12].

### 2.3 DNA Extraction, Sequencing and Analysis

DNA was extracted from freshly grown colonies according to the ZR Fungal/Bacterial DNA MiniPrep (Zymo Research, Irvine, CA, USA) kit, with one modification. The final elution buffer was replaced for one lacking EDTA. This allowed the DNA to be eligible for sequencing by the standards set by MicrobesNG. DNA was sent for whole‐genome Illumina sequencing at 90× coverage and sequence assembly to MicrobesNG, University of Birmingham, UK. Sequences were deposited in the NCBI database under BioProject ID: PRJNA1454038.

#### 2.3.1 Single‐Nucleotide Polymorphism Detection

SNPs of the ATCC *C. tropicalis* MYA‐3404 genomes were compared to the SNPs of the isolates by using the snippy multicommand (snippy‐base application v4.5.0) [20] that generates a core genome multiple alignment against a common reference. The ATCC *C. tropicalis* was used as a reference. The pipeline detects the variants and generates a single file for each isolate, listing the different variations. Snippy (snippy‐multi, snippy‐core; v4.5.0), uses BWA‐MEM for read alignment and FreeBayes as the variant caller in haploid mode. SNPs were called at sites with a minimum depth of 10 reads, minimum Phred‐scaled variant quality of 100 and minimum mapping quality of 60; positions not meeting these thresholds were treated as missing data and excluded from downstream analyses. Because Snippy is optimised for haploid bacterial/yeast genomes, only high‐confidence homozygous alternative alleles relative to the reference were retained in the core SNP alignment and putative heterozygous sites (mixed allele calls) were ignored. SNP tables were then combined with snippy‐core to obtain a core‐genome alignment, and only biallelic SNPs present in all isolates (core SNPs) were used for gene‐level analyses. Genes involved in resistance, biofilm formation, plasma membrane, cell walls, virulence, adhesion and other characteristics were analysed. These genes are summarised in Table 1.

**TABLE 1:** Genes analysed for SNP mutations grouped by similar functions.

Function	Genes
Adhesion & biofilm formation	*ALS1, ALS2, WOR1, ALS6, EFG1, HWP1*
Transcriptional regulators	*CPH1, CPH2, MLT1*
Drug efflux pumps/multidrug resistance	*CDR1, CDR2, CDR3, CDR4, MDR1, MMR1, SNQ2, TAC1*
Cell wall biosynthesis	*CHS1, CHS3, FKS1, FKS2*
Virulence factors	*SAP3, SAP7, SAP9*
Ergosterol biosynthesis pathway	*ERG2, ERG3, ERG4, ERG6, ERG7, ERG8, ERG9, ERG11, ERG12, ERG20, ERG25, ERG26, UPC2*
DNA repair	*MSH3, MSH4*

*Note:* Not all genes exhibited mutations compared to the control strain. The GenBank accession numbers for all genes are found in Table S1.

#### 2.3.2 Annotation

Gene prediction was achieved using the BRAKER2 v2.1.6 pipeline in fungus mode, which combines GeneMarK‐ES v4.65 and AUGUSTUS 3.4.0 for fungal gene prediction and identifying gene locations with the corresponding CDS and messenger RNA (mRNA) qualifiers [21, 22, 23]. Interproscan 5.50–84.0 [24] was used on the COG database to create the xml file to further incorporate in the functional annotation pipeline created by Funannotate 1.8.7 [25, 26]. The pipeline starts by running HMMscan (HMMer v3.3) (hmmer.org) with default parameters on the PFAM database, then using emapper 2.1.2 based on eggnog orthology data [27]. Sequence searches were performed using Diamond Blastp v.0.4.7 [28] on UniProt DB version 2021_02 and MEROPS v12.0; the resulting annotations were combined using Gene2Product v1.69, and later, Signalp 5.0 was used to predict secreted proteins [29]. Assemblies and annotations were assessed using BUSCO V5.2.2 [30].

#### 2.3.3 Phylogenetic Analysis

To determine the phylogenetic relationship between the isolates, the core genome sequence, recombination data and single nucleotide polymorphisms (SNPs) in conjunction with parsnp v1.2, a tool available in the Harvest suite was utilised. The corresponding reference genome was used for this analysis. SNPs identified in local collinear blocks were then employed to construct an approximate maximum‐likelihood tree using FastTree, incorporating the general time reversible (GTR) model of nucleotide substitution. We applied the Shimodaira–Hasegawa test, implemented in FastTree2, to evaluate the support for significant clustering observed in the phylogenetic tree. For graphical representation and annotation, we utilised the interactive tree of life (iTOL) [31, 32].

#### 2.3.4 Pairwise Genome Similarity Heatmap

Pairwise genome similarity heatmap based on Mash‐derived distances by Stevens et al. [33]. Genetic relatedness among the genomes was compared using Mash. Cell represents pairwise similarity expressed as either Mash distance or converted to approximate Average Nucleotide Identity (ANI) between two genomes, which was performed using R statistical computing software. ANI measures the genomic similarity between two organisms.

### 2.4 Quantification of Ergosterol in the Plasma Membrane

A single colony of each yeast isolate was grown in 50 mL potato dextrose broth (PDB) for 18 h at 35°C at 100 rpm. Extraction and quantification of ergosterol was done as outlined in [34] with one modification: isolates were boiled at 85°C for 2 h. Optical density measurements at 230 and 281.5 nm were taken by a Genesys 10S UV–Vis spectrophotometer (Thermo Scientific, Waltham, Massachusetts, USA) in glass quartz cuvettes. The experiment for each isolate was performed in biological triplicates. The average percent ergosterol (erg.) was computed and compared to the control ATCC *C. tropicalis* isolate by the formula:

$$\%\text{change in}\;\mathrm{erg}.\;\text{content}=\left[\left(\%\mathrm{erg}.\;\text{in isolate}-\%\mathrm{erg}.\;\text{in ATCC}\right)/\%\mathrm{erg}.\;\text{in ATCC}\right]\times 100$$

### 2.5 Quantification of Rhodamine 6 g Efflux

Rhodamine 6G (R6G) efflux by yeast efflux pumps was quantified as outlined in [35]. Colonies were grown in 10 mL PDB overnight at 32°C at 100 rpm. 5.6 × 10^8^ cells of *C. tropicalis* were pelleted by 5 min centrifugation at 3000 rpm. Cells were starved and suspended for 2 h in 10 mL of glucose‐free 1× phosphate buffered saline solution (PBS) while shaking at 32°C at 100 rpm. A final concentration of 10 μM of R6G was added to the cells and were put back in the incubator for 1 h. Cells were washed with glucose‐free 1× PBS once and then resuspended in 10 mL of 1× PBS + 2% glucose for another 1 h in the incubator. During this last incubation period, 1.5 mL of the supernatant was taken at 15, 30, 45 and 60 min and centrifuged at 9000 *g* in a microcentrifuge. The optical density of 1 mL of supernatant was measured at 527 nm, using a Genesys 10S UV–Vis spectrophotometer (Thermo Scientific, Waltham, Massachusetts, USA). The experiment was performed in biological triplicates.

### 2.6 Adhesion to Human Epithelial Cells

Adherence to human epithelial cells was tested as described in [36]. A 6‐well microtiter plate was incubated with the human epithelial cell line, SK_OV‐3. Yeast isolates were grown overnight in PDB at 37°C and 100 rpm. About 100 cells were incubated with the human cells for 90 min. Wells were washed 3 times with 1× PBS to remove non‐adherent Candida cells and then overlain with molten PDA. The number of colonies was counted and compared to control wells that were not washed. The experiment was performed in biological triplicates.

### 2.7 Crystal Violet Biofilm Assay

Biofilm production was quantified as described in [37] with some modifications. 10^7^ cells/mL were suspended in 150 μL PDB and inoculated in 96‐well plates, which were incubated at 37°C and 90 rpm for 48 h at 37°C. The PDB was thoroughly discarded and the biofilms were fixed with 200 μL of pure methanol for 15 min. After discarding methanol, the plates were air dried for 20 min and stained with 150 μL of 1% crystal violet for 3 min. The plates were washed five to six times with distilled water until the water ran clear. Crystal violet was solubilised with 150 μL of 33% acetic acid in each well. Optical densities were measured after 15 min and moved to a clean 96‐well plate at 595 nm using the Multiskan FC Microplate Photometer (Thermo Fisher Scientific, Rockford, IL, USA). The experiment was performed in biological triplicates for each isolate.

### 2.8 Chitin Quantification Assay

Cells were grown in 5 mL of PDB overnight in a shaking incubator at 30°C at 100 rpm. Cells were pelleted by 5 min centrifugation at 4000 rpm. Pellets were resuspended with 500 μL of 5 mM Tris (pH 7.8) and moved into a 2 mL Eppendorf tube with three sterile, cold glass beads of and vortexed for 1 h. The mixtures were poured into a new 1.5 mL pre‐weighed Eppendorf tubes and the beads were washed with 50 μL 1 M NaCl, which was added to 1.5 mL tubes. The tubes were centrifuged at 3000 rpm for 5 min and the pellets were weighed. 0.5 mL of protein extraction buffer (150 mM NaCl, 100 mM Na‐EDTA, 50 mM Tris buffer, 2% SDS, 8 μL/1 mL β‐mercaptoethanol, pH 7.8) was added for every 100 mg of pellet for resuspension and the mixture was boiled for 10 min at 99°C, let to cool for 5 min and then centrifuged for 5 min at 3000 room. This step was performed twice. Pellets were then washed by resuspension in sterile water three times and centrifuged for 5 min at 3000 rpm. Pellets were resuspended in 1 mL of 6 N HCl and boiled for 10 min at 99°C, centrifuged and resuspended in 1 mL of sterile water. Quantification of cell wall chitin was performed as the protocol described in [38]. The experiment was performed in biological triplicates.

### 2.9 Virulence Assay

Virulence was measured through systemic infection in murine models of disseminated candidiasis [39]. Isolates were grown overnight in 10 mL PDB at 30°C in a shaking incubator at 100 rpm. 4 × 10^8^ cells were resuspended in 0.2 mL of 1× PBS solution and injected into the tail vein of 4 to 6‐week‐old BALB/c female mice. Injected mice were monitored for 30 days and the number of moribund mice was counted daily. A negative control group of 6 mice were injected with 1× PBS without yeast cells. Moribund mice were euthanised.

### 2.10 Statistical Analysis

All figures were generated on GraphPad Prism version 8.0.2. For the ergosterol, chitin, biofilm and human‐adhesion assays, statistical significance was assessed by one‐way analysis of variance (ANOVA) followed by Dunnett's multiple comparison test. For the virulence assay, statistical significance was determined using the log‐rank (Mantel‐Cox) test. For the R6G efflux assay, an ordinary two‐way ANOVA test was used. *p* values less than or equal to 0.05 meant the data were statistically significant.

### 2.11 Ethics Statement

This study was performed after the approval by the Institutional Review Board of the Lebanese American University on December 222,023 under IRB# LAU.ACUC.SAS.RK1.22/December/2023. Protocols followed the ethical standards of the Lebanese American University's Institutional Animal Care and Use Committee. The study was conducted under the approval number: LAU.ACUC.SAS.RK1.22/December/2023.

## 3 Results

### 3.1 *Candida tropicalis* Identification

Of the twenty‐five hospital isolates labelled as *C. tropicalis* , twenty‐two were identified as *C. tropicalis* according to growth on CHROMagar media. *C. tropicalis* gives a distinctive deep, dark blue to purple colour [40]. These isolates were then sent for whole genome sequencing, which revealed that only fourteen isolates were truly *C. tropicalis* . Two isolates were *C. glabrata* , one was *C. sojae* and three isolates were cocultures of *C. tropicalis* and *C. sojae*. The remaining three were a coculture of multiple other *Candida* species.

### 3.2 Antifungal MIC Determination

As can be seen in Table 2 all 14 hospital isolates were tested for susceptibility to various antifungal agents and determined susceptible, intermediate or resistant according to the Clinical and Laboratory Standards Institute (CLSI) M60 guidelines. All isolates were resistant to fluconazole, a first line agent used to treat candida infection [19], except for isolate 50, which was susceptible dose‐dependent. Both isolates 50 and 57 were resistant to posaconazole. Isolate 58 was the only isolate resistant to both an azole (fluconazole) and an echinocandin (micafungin).

**TABLE 2:** Antifungal susceptibility profiles of *C. tropicalis* hospital isolates expressed in μg/mL.

Isolate #	FLC	POS	VOR	CAS	MIC	AND
46	64	< 0.03125	< 0.03125	< 0.03125	< 0.03125	< 0.03125
47	16	< 0.03125	< 0.03125	0.0625	< 0.03125	< 0.03125
50	*4*	0.5	< 0.03125	< 0.03125	< 0.03125	< 0.03125
52	64	< 0.03125	< 0.03125	< 0.03125	< 0.03125	< 0.03125
53	64	< 0.03125	< 0.03125	< 0.03125	< 0.03125	< 0.03125
54	8	< 0.03125	0.0625	0.0625	< 0.03125	0.0625
57	64	16	< 0.03125	< 0.03125	0.125	< 0.03125
58	64	< 0.03125	< 0.03125	< 0.03125	2	< 0.03125
60	64	< 0.03125	< 0.03125	< 0.03125	< 0.03125	< 0.03125
61	16	< 0.03125	< 0.03125	< 0.03125	< 0.03125	< 0.03125
62	64	< 0.03125	< 0.03125	< 0.03125	< 0.03125	< 0.03125
63	64	< 0.03125	< 0.03125	< 0.03125	< 0.03125	< 0.03125
65	64	< 0.03125	< 0.03125	< 0.03125	< 0.03125	< 0.03125
66	16	< 0.03125	< 0.03125	< 0.03125	< 0.03125	< 0.03125

*Note:* Minimum inhibitory concentrations (MIC) values that are underlined denote resistance and values in italics are intermediates.

Abbreviations: AND, anidulafungin; CAS, caspofungin; FLC, fluconazole; MIC, micafungin; POS, posaconazole; VOR, voriconazole.

### 3.3 Sequencing Analysis

**FIGURE 1:**
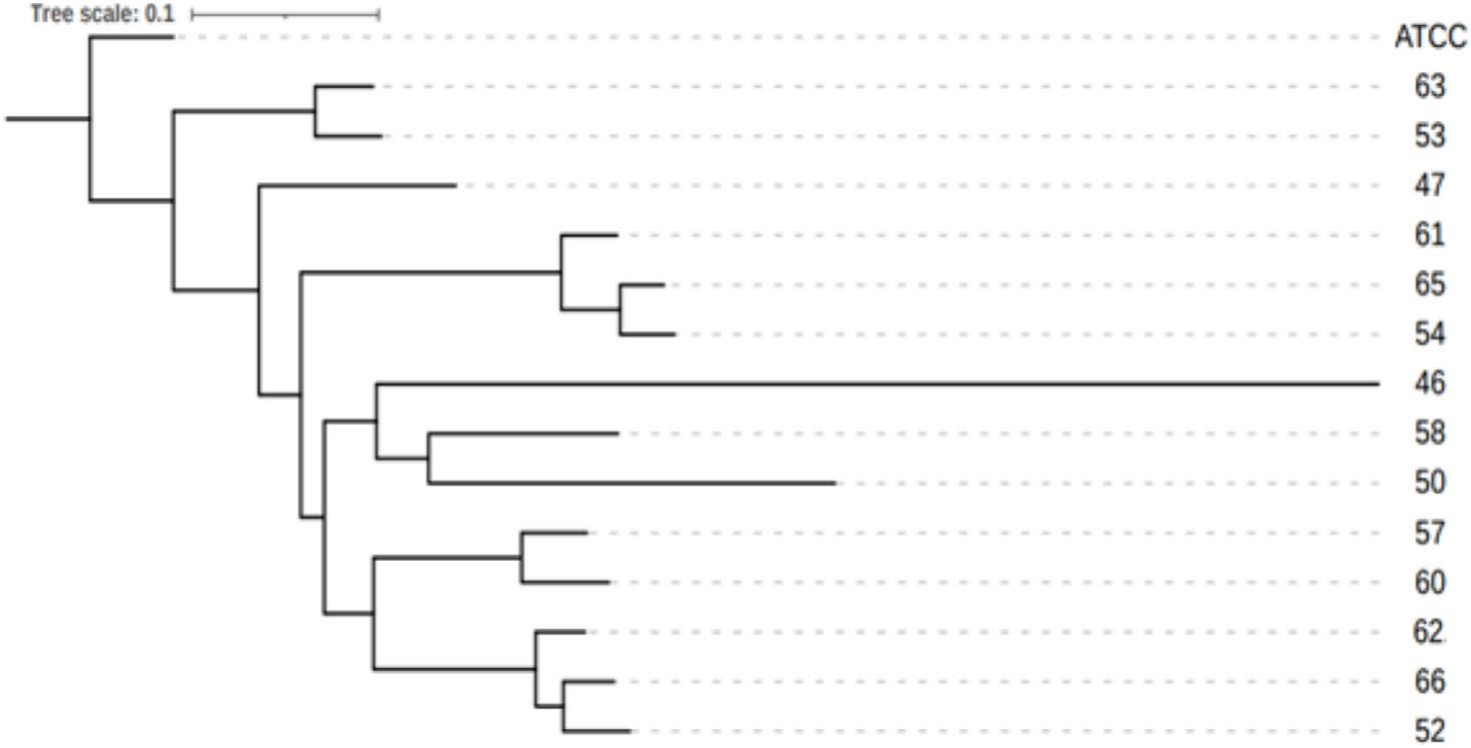
SNP‐based phylogenetic tree of hospital isolates and the ATCC control reference genome, *C. tropicalis* MYA‐3404.

**FIGURE 2:**
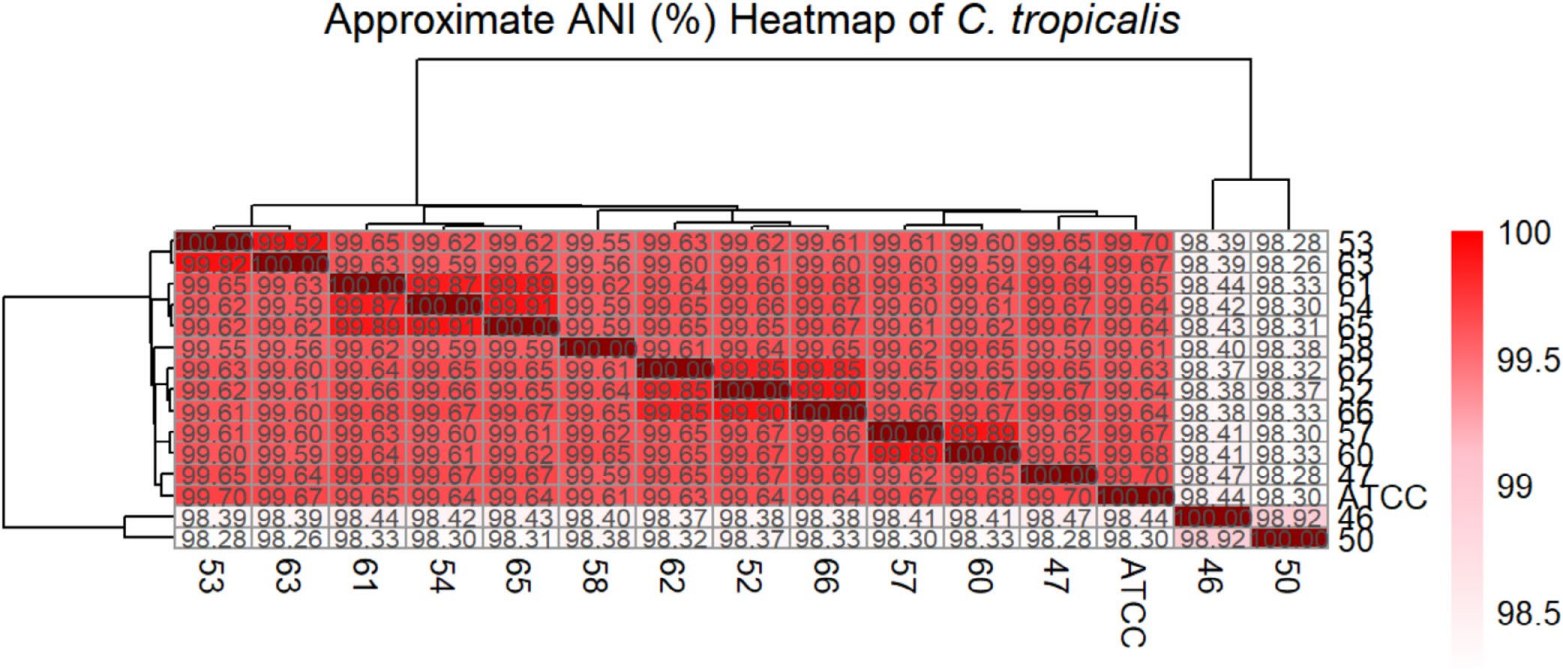
Heat map analysis of all fourteen hospital *C. tropicalis* isolates. Each cell represents the ANI value in percentage calculated between two genomes. The colour of each cell ranges from light to dark red, corresponding to lower and higher ANI values, respectively.

The genomes of all *C. tropicalis* hospital isolates were analysed for SNP mutations as can be seen in Table 3 for mutations in key genes and Table S2 for mutations in the remaining genes. The overall genomes were compared to the control strain to generate a SNP based phylogenetic tree (Figure 1), pairwise heat‐map (Figure 2) and a table of chromosomal changes (Table 4).

**TABLE 3:** Amino acid substitutions detected in key genes in the *C. tropicalis* hospital isolates compared to the ATCC control strain, *C. tropicalis* MYA‐3404.

	Isolate 46	Isolate 47	Isolate 50	Isolate 52	Isolate 53	Isolate 54	Isolate 57	Isolate 58	Isolate 60	Isolate 61	Isolate 62	Isolate 63	Isolate 65	Isolate 66
*CDR1*	Ile96Val	—	Ile96Val, Pro209Ser, Thr233Ser, Pro217His, His305Asp	Pro209Ser, Thr233Ser, His305Asp, Met15Val, Lys138Asn	Gly102Arg, Arg307Gln, Leu89Gln, Gln93Leu, Pro96Leu	Pro209Ser, Thr233Ser, Lys138Asn, Lys332Asn	Pro209Ser, Thr233Ser, His305Asp	Pro209Ser, Thr233Ser, Pro60Arg	Pro209Ser, Thr233Ser, His305Asp, Pro60Arg	Pro209Ser, Thr233Ser, Lys138Asn, Lys332Asn	Pro209Ser, Thr233Ser, His305Asp, Lys138Asn, Cys202Tyr, Arg17Gln	Gly102Arg, Arg307Gln, Leu89Gln, Gln93Leu, Pro96Leu	Pro209Ser, Thr233Ser, Lys332Asn	Pro209Ser, Thr233Ser, His305Asp, Met15Val, Lys138Asn, Ile37Val, Lys297Gln, Met283Ile, Ser253Pro, Ile170Val, Ile67Thr, Arg58Leu
*CDR2*	Asn50Lys, Glu291Lys	Glu291Lys, Thr694Ile	Glu291Lys, Arg9Lys	Glu291Lys	Glu291Lys, Gly469Asp	Val117Met, Thr561Ala	Glu291Lys	Glu291Lys, Ile205Val	Glu291Lys	Thr561Ala	Glu291Lys, Thr694Ile, Val117Met	Glu291Lys	—	Glu291Lys, Val117Met, Thr561Ala
*CDR3*	Ala16Thr, Glu578Lys, Ser55Asn	Ala16Thr	Ala16Thr	Ala16Thr	—	Glu578Lys, Ser55Asn	Ala16Thr	Ala16Thr	Ala16Thr	—	Ala16Thr, Glu578Lys, Ser55Asn	Ala16Thr	Ala16Thr, Glu578Lys, Ser55Asn	Ala16Thr
*CDR4*	Thr43Ile, stop_gained Ser223*, Ser416Asn, Gly469Asp	—	Thr43Ile, stop_gained Ser223*, Val71Gly	Met757Thr	—	Met757Thr	Met757Thr	—	Met757Thr, Thr293Met	Met757Thr	—	Ala521Val	—	Thr293Met, Phe537Ser
*ERG11*	—	—	Asp340Ala, Glu337Asp, Tyr130Phe	—	—	—	—	—	—	—	—	—	—	—
*ERG20*	Lys314Glu	Lys314Glu	Lys314Glu	Lys314Glu	Lys314Glu	Lys314Glu	Lys314Glu	Lys314Glu	Lys314Glu	Lys314Glu	Lys314Glu	Lys314Glu	Lys314Glu	Lys314Glu
*CHS1*	—	—	—	Asp143Glu	—	—	—	Asp143Glu	Asp143Glu	Asp143Glu	Asp143Glu	—	Asp143Glu	Asp143Glu
*CHS3*	Gln38Leu	—	Gln38Leu, Leu715His	—	—	—	—	Pro158Ser, Glu96Asp	—	—		—	Lys163Asn	—
*FKS1*	—	—	Asp917Glu, Ile1156Thr, Val1352Ile, Val1404Ile	—	—	—	—	—	—		—	—	Gly25Val	—

**TABLE 4:** Chromosomal changes found in all fourteen hospital *C. tropicalis* isolates.

Isolate	Complex substitutions	Deletions	Insertions	MNPs	SNPs	Total chromosomal changes
46	5799	1980	2071	12	26,510	36,372
47	1291	2226	2184	4	13,172	18,877
50	2716	1363	1362	19	15,829	21,289
52	1715	2589	2620	3	16,464	23,391
53	1173	1929	2012	2	11,395	16,511
54	2276	3102	3200	4	20,873	29,455
57	1789	2681	2524	1	15,300	22,295
58	1692	2268	2343	9	15,409	21,721
60	1933	2608	2638	6	16,865	24,050
61	1831	2784	2741	3	17,483	24,842
62	2047	3145	2913	4	18,955	27,064
63	1053	1714	1819	—	10,545	15,131
65	1980	2725	2890	2	18,577	26,174
66	1499	2212	2288	1	15,448	21,448

*Note:* Differences are relative to the control strain.

Abbreviations: MNP, multiple‐nucleotide polymorphisms; SNP, single‐nucleotide polymorphisms.

### 3.4 Quantification of Ergosterol in the Plasma Membrane

**FIGURE 3:**
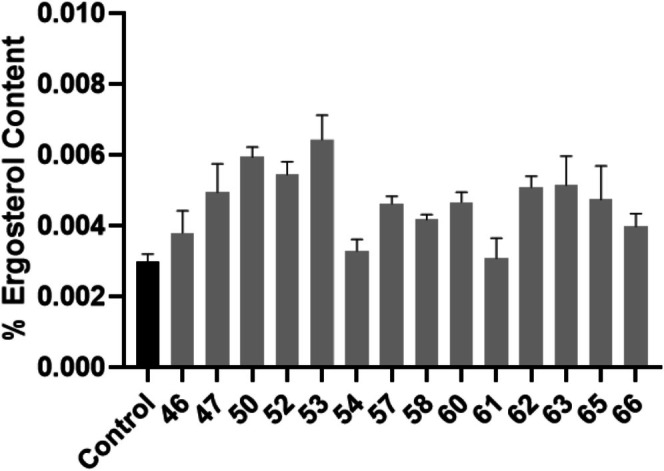
Quantification of ergosterol content. The black bar represents the control strain *C. tropicalis* MYA‐3404, that is sensitive to antifungal agents. The grey bars represent the hospital isolates. Statistical significance was calculated using One‐way ANOVA (*p* < 0.0001) coupled to Dunnett's multiple comparisons test: Control versus 46: *p* = 0.4182; Control versus 47: *p* = 0.0009; Control versus 50: *p* < 0.0001; Control versus 52: *p* < 0.0001; Control versus 53: *p* < 0.0001; Control versus 54: *p* = 0.9957; Control versus 57: *p* = 0.0066; Control versus 58: *p* = 0.0838; Control versus 60: *p* = 0.0051; Control versus 61: *p* = 0.9997; Control versus 62: *p* = 0.0004; Control versus 63: *p* = 0.0003; Control versus 65: *p* = 0.0032; Control versus 66: *p* = 0.2012.

The major sterol present in the fungal plasma membrane is ergosterol. Changes to the sterol, either the content or the amount produced, are a well‐known effect in azole‐resistance [19]. An overall increase in ergosterol content was observed in hospital isolates compared to the control strain (Figure 3). Only isolates 46, 54, 58, 61 and 66 did not show a significant increase in ergosterol production.

### 3.5 Quantification of Rhodamine 6G Efflux

**FIGURE 4:**
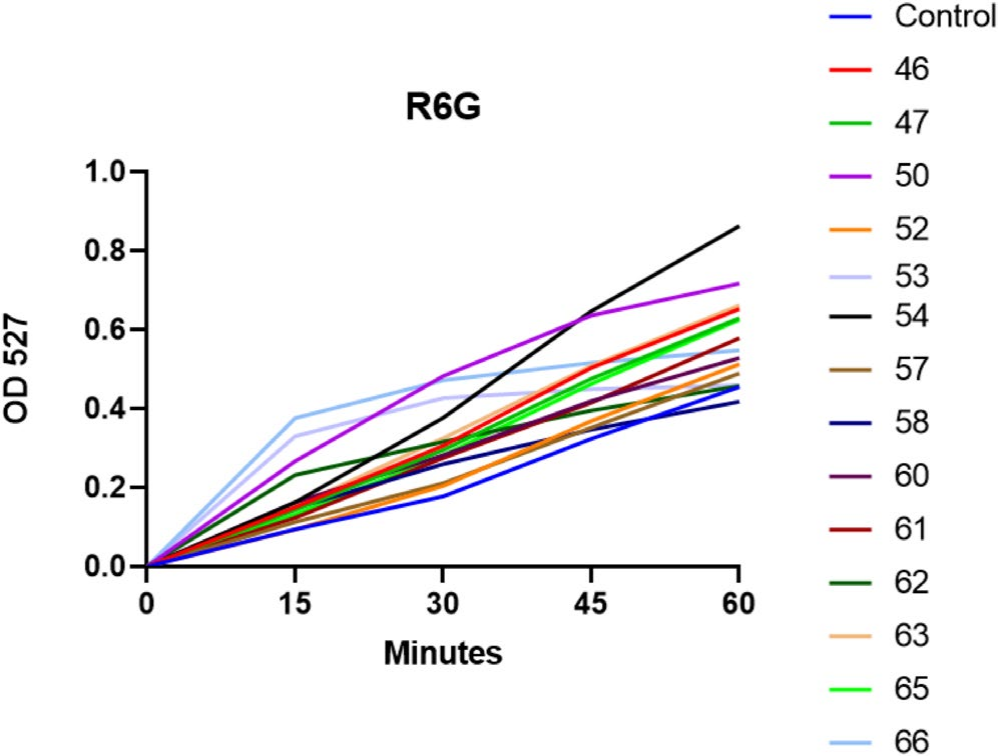
Quantification of R6G efflux over time. The bright blue line is the control isolate. All other hospital isolates are denoted by the nearby legend. Statistical significance was calculated using two‐way ANOVA (*p* < 0.0001) coupled to Dunnett's multiple comparisons test: Control versus 46: *p* = 0.001; Control versus 47: *p* = 0.0042; Control versus 50: *p* < 0.0001; Control versus 52: *p* = 0.947; Control versus 53: *p* = 0.0003; Control versus 54: *p* < 0.0001; Control versus 57: *p* = 0.9777; Control versus 58: *p* = 0.9658; Control versus 60: *p* = 0.083; Control versus 61: *p* = 0.083; Control versus 62: *p* = 0.0713; Control versus 63: *p* = 0.0005; Control versus 65: *p* = 0.0104; Control versus 66: *p* < 0.0001.

R6G was used to quantify the upregulation of efflux‐pump expression. These pumps are used by the yeast to expel internalised substances, such as antifungal drugs. Increased expression of efflux pumps confers resistance against antifungal agents [41]. Hospital isolates exhibited an increase in expression of efflux pumps (Figure 4); isolates 54 and 50 had the greatest increase at 60 min compared to the control. Isolates 52, 57, 58, 60, 61 and 62 did not show a significant difference in R6G output.

### 3.6 Adhesion to Human Epithelial Cells

**FIGURE 5:**
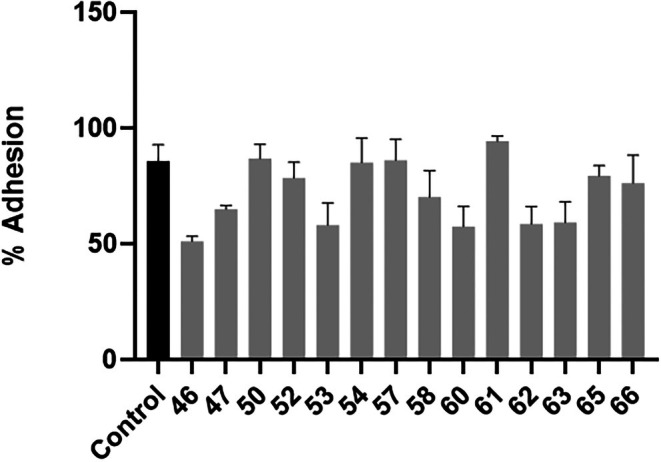
Adhesion to human cells. The black bar represents the control strain *C. tropicalis* MYA‐3404, that is sensitive to antifungal agents. The grey bars represent the hospital isolates. Statistical significance was calculated using One‐way ANOVA (*p* < 0.0001) coupled to Dunnett's multiple comparisons test: Control versus 46: *p* = 0.0001; Control versus 47: *p* = 0.0283; Control versus 50: *p* = 0.9997; Control versus 52: *p* = 0.9341; Control versus 53: *p* = 0.002; Control versus 54: *p* = 0.9999; Control versus 57: *p* > 0.9999; Control versus 58: *p* = 0.1689; Control versus 60: *p* = 0.0016; Control versus 61: *p* = 0.8017; Control versus 62: *p* = 0.0027; Control versus 63: *p* = 0.0035; Control versus 65: *p* = 0.9653; Control versus 66: *p* = 0.6953.

Adherence plays a major part in Candida virulence, as it is the first interaction between the pathogen and host tissue. This is largely mediated by cell wall adhesins such as the *ALS* and *HWP* family of proteins [42]. As can be seen in Figure 5, there was an overall decrease in adhesion seen among the hospital isolates. Isolates 46, 47, 53, 58, 60, 62 and 63 were the only isolates significantly less adherent compared to the control isolate.

### 3.7 Crystal Violet Biofilm Assay

**FIGURE 6:**
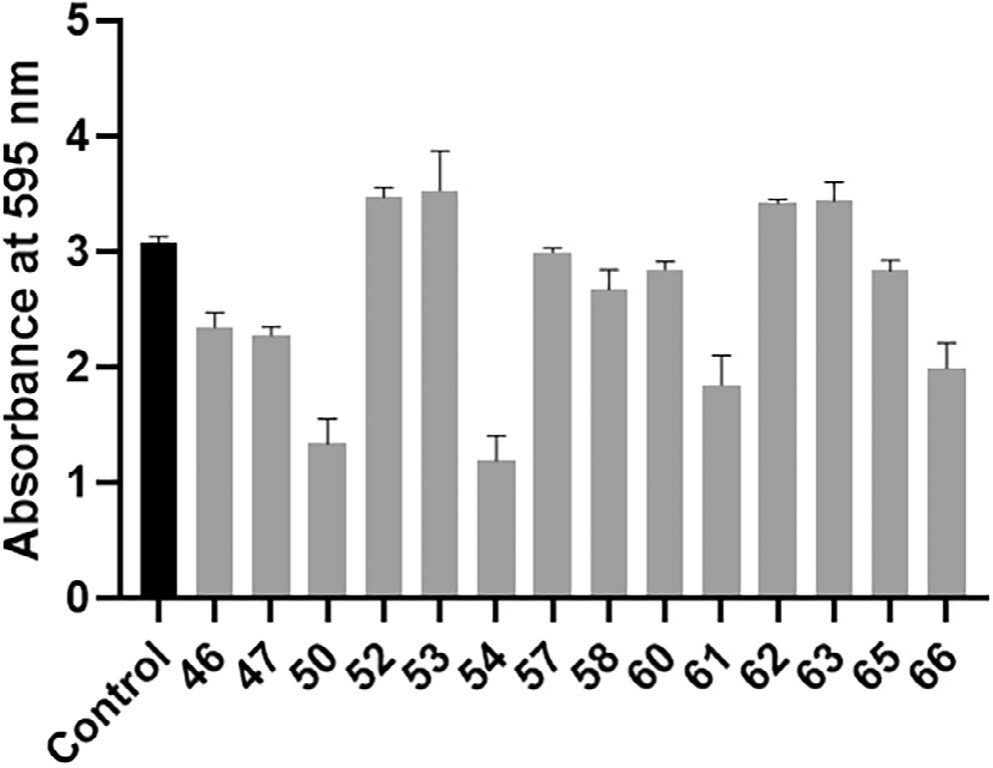
Biofilm formation. The black bar represents the control strain *C. tropicalis* MYA‐3404, that is sensitive to antifungal agents. The grey bars represent the hospital isolates. Statistical significance was calculated using one‐way ANOVA (*p* < 0.0001) coupled to Dunnett's multiple comparisons test: Control versus 46: *p* = 0.0001; Control versus 47: *p* < 0.0001; Control versus 50: *p* < 0.0001; Control versus 52: *p* = 0.0588; Control versus 53: *p* = 0.0221; Control versus 54: *p* < 0.0001; Control versus 57: *p* = 0.9993; Control versus 58: *p* = 0.0644; Control versus 60: *p* = 0.5937; Control versus 61: *p* < 0.0001; Control versus 62: *p* = 0.1254; Control versus 63: *p* = 0.0971; Control versus 65: *p* = 0.5359; Control versus 66: *p* < 0.0001.

Biofilm formation of isolates showed diversity among the clinical isolates compared to the control (see Figure 6). Isolate 53 was the only isolate to show significant higher biofilm production, while isolates 46, 47, 50, 54, 61 and 66 showed significantly lower biofilm production. All other isolates had a crystal violet output similar to that of the control. *C. tropicalis* are known as strong biofilm producers among the *Candida* species [9, 11].

### 3.8 Quantification of Cell Wall Chitin

**FIGURE 7:**
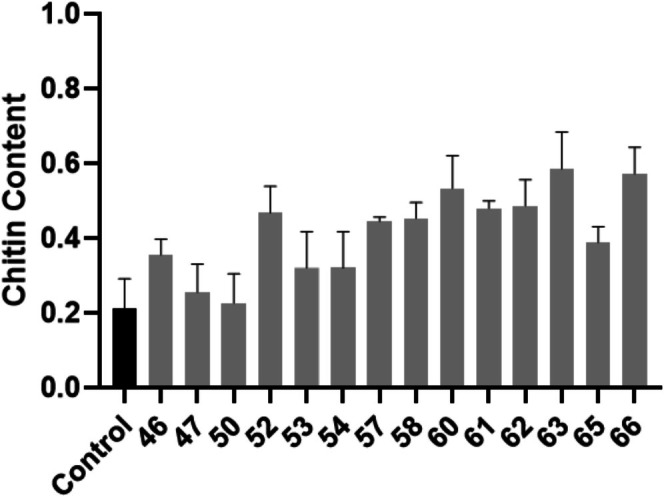
Quantification of chitin content. The black bar represents the control strain *C. tropicalis* MYA‐3404, that is sensitive to antifungal agents. The grey bars represent the hospital isolates. Statistical significance was calculated using One‐way ANOVA (*p* < 0.0001) coupled to Dunnett's multiple comparisons test: Control versus 46: *p* = 0.1409; Control versus 47: *p* = 0.9911; Control versus 50: *p* = 0.9996; Control versus 52: *p* = 0.0012; Control versus 53: *p* = 0.408; Control versus 54: *p* = 0.408; Control versus 57: *p* = 0.0034; Control versus 58: *p* = 0.0024; Control versus 60: *p* < 0.0001; Control versus 61: *p* = 0.0007; Control versus 62: *p* = 0.0005; Control versus 63: *p* < 0.0001; Control versus 65: *p* = 0.0403; Control versus 66: *p* < 0.0001.

Chitin content in hospital isolates was overall significantly higher than in the control isolate (Figure 7). In resistant isolates, chitin content in the cell wall tends to increase specifically in cases of resistance to echinocandins [43]. Isolate 58 was the only isolate with confirmed resistance to micafungin and displayed a 153% increase in chitin content. Isolates 46, 47, 50, 53 and 54 did not show a significant increase in chitin production.

### 3.9 Virulence Assay

**FIGURE 8:**
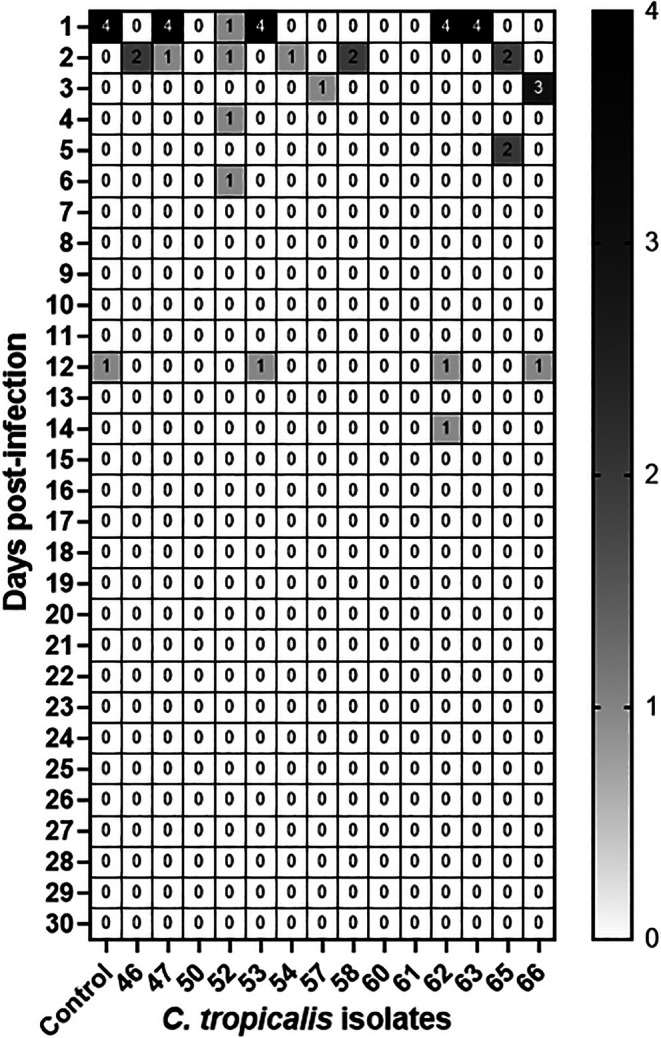
Heat‐map of virulence potential in murine disseminated model of infection. Six BALB/c mice were injected with each isolate and monitored for survival over 30 days. The number of mouse deaths per day and per isolate is shown in the colour‐coded cells. Logrank test: Control versus 46: *p* = 0.2499; Control versus 47: *p* > 0.9999; Control versus 50: *p* = 0.0229; Control versus 52: *p* = 0.7349; Control versus 53: *p* > 0.9999; Control versus 54: *p* = 0.0976; Control versus 57: *p* = 0.2499; Control versus 58: *p* = 0.2499; Control versus 60: *p* = 0.0229; Control versus 61: *p* = 0.0229; Control versus 62: *p* > 0.9999; Control versus 63: *p* = 0.7271; Control versus 65: *p* = 0.7356; Control versus 66: *p* = 0.7342.

A wide range of virulence was seen from the injected isolates, as seen in Figure 8. Three isolates, 50, 60 and 61, had no deaths by the end of the thirty‐day period, a significant difference compared to the control isolate. All other isolates displayed virulence similar to that of the control isolate. *C. tropicalis* is often associated with higher mortality and morbidity rates compared to other *Candida* species [4, 5].

## 4 Discussion

Candidiasis infections by non‐*albicans Candida* species continue to appear more frequently in global populations [4]. The healthcare system in Lebanese hospitals generally relies on CHROMagar or morphological identification, which has its own limitations. Our lab had previously sequenced samples from supposed *C. parapsilosis* hospital infections, but found two out of eight isolates were misidentified [44]. Similarly, the isolates sampled for this study showed a significant case of misidentification, even after confirmation by streaking on CHROMagar. Of the 25 isolates initially identified as *C. tropicalis* , whole‐genome sequencing confirmed only 14 (56%) as pure *C. tropicalis* . Three additional isolates (12%) contained *C. tropicalis* in mixed‐species cultures, bringing the total number of isolates in which *C. tropicalis* was detected to 17 (68%). The remaining eight isolates (32%) did not contain *C. tropicalis* . Given that hospitals cannot be realistically asked to determine species identity by genome sequencing or mass spectrometry, misidentification is to be expected. Despite numerous similarities, non‐*albicans Candida* species do not have similar responses and resistance to antifungal agents; certain species exhibit intrinsic resistance to common first‐line antifungal agents, such as *C. glabrata* and *C. krusei* to fluconazole [45, 46]. Proper treatment for candidiasis will require efficient identification of the fungal pathogen. The extensive use of fluconazole has particularly contributed to the increase in azole resistant *Candida* species, including *C. tropicalis* . *C. glabrata* shows the highest rates of fluconazole‐acquired resistance when compared to all other *Candida* species, but *C. tropicalis* overtakes *C. albicans* in this regard. The rate of fluconazole resistant *C. tropicalis* is especially high in South Asian countries, such as China, India and Vietnam, as well as Austria [47].

Our study aimed to investigate the molecular mechanism of resistance and pathogenicity. This was done by analysing genes of interest for amino acid substitutions in our *C. tropicalis* isolates and characterising their phenotypic attributes. All samples were compared to a control strain, *C. tropicalis* MYA‐3404, that is susceptible to all known antifungal drugs. All isolates were resistant to at least one of two azole drugs, fluconazole and posaconazole. Isolate 57 was the only isolate to display resistance to both azoles. Currently, azole resistance is an emerging phenomenon occurring in numerous countries [4, 47]. Isolate 58 was the only isolate exhibiting double resistance to an azole and an echinocandin, specifically micafungin. Although not as common as azole resistance, echinocandin resistance is also increasingly being reported on [4, 48]. Development of novel antifungal agents is limited and slow; the yeast species is eukaryotic; therefore, drugs that target only yeast cells without harming patient cells are difficult to discover [49]. Consequently, the appearance of doubly resistant isolates is concerning.

Our generated phylogenetic tree indicates that the isolates are relatively closely related but do separate into several distinct clades. SNP analysis revealed distinct but shared mutation profiles. All isolates exhibited the Lys314Glu mutation in the *ERG20* gene, which suggests a common polymorphism that provides a baseline fitness advantage in the hospital setting. Additionally, many isolates displayed numerous shared mutations in the efflux‐drug pumps *CDR1*, *CDR2*, *CDR3* and *CDR4*. For example, the *CDR2* mutation Glu291Lys appeared in eleven isolates and the *CDR3* mutation Ala16Thr appeared in another eleven isolates. The combination of the Pro209Ser and Thr233Ser in *CDR1* appeared in nine isolates, often accompanied with His305Asp or Lys138Asn. This shows a clear repeated patten of mutations in the drug‐efflux pumps. The presence of mutations in *CDR1* in resistant *C. albicans* has been before reported before [50]. *C. tropicalis* has so far shown that over‐expression of efflux pumps is highly tied to resistance [19]. Similar mutations are found in other genes across a few isolates but others show distinct mutations. Average nucleotide identity analysis revealed that the isolates are closely related, with ANI values ranging from 99.6% to 99.9%. This could indicate a very recent common ancestor within the hospital environment; however, the isolates exhibit divergence through a limited amount of point mutations. Coupled with the phylogenetic tree, the isolates show distinct clustering. For example, isolates 53 and 63 cluster together. The two isolates share the same set of point mutations in the *CDR1* gene, as well as a few other mutations in genes involved in the ergosterol pathway and chitin production. Interestingly, the isolates showed similar results in only the adhesion and biofilm assay. This is likely due the presence of other unique mutations. Isolates 54 and 65 also cluster together, as well as 52 and 66. Despite numerous shared mutations within these clusters, the presence of unique mutations again likely influences the differing phenotypic characteristics. Isolates 46 and 50 stand out as genomic outliers; the two isolates occupy the longest branches on the phylogenetic tree and contain unique mutations. Isolate 46 has the most total chromosomal changes of all the other isolates. Both isolates gained a stop codon at position Ser223* in *CDR4*. Isolate 46 displayed the largest drop in adhesion while isolate 50 had the second highest output of R6G. Overlaying the mutational analysis with the phylogenetic tree and ANI heatmap highlights evidence of a closely related population of *C. tropicalis* that has adapted to the hospital environment and accumulated a conserved set of resistance‐associated mutations. The high genomic similarity and shared mutational profiles raise the possibility of localised clonal expansion, although epidemiological data were not available to assess transmission dynamics. Outbreak cannot be ruled out given how closely some of the isolates cluster together and the low level of genomic divergence among them. Small genomic differences could be due to strain microevolution within the hospital ward resulting in the very slightest divergence observed. *C. tropicalis* generally shows high genetic diversity based on multilocus sequence typing but certain regional populations show phylogenetic similarity due to shared ancestry [51]. Outbreaks have previously been reported, particularly in neonatal and adult ICUs [9]. The study noted that a potential major source of outbreaks could be cross‐contamination from hand carriage by healthcare workers.

Resistance to azoles in *Candida* species occurs in a multitude of methods; the yeast species could increase the overall ergosterol content in its cell membrane to offset the effects of the azole drug, change the composition of its sterol content to an intermediate instead of the drug target or upregulate the production of efflux drug pumps to physically remove the drug. Our isolates showed an overall increase in ergosterol, as well as an upregulation in efflux‐pumps. *C. tropicalis* has demonstrated a correlation between fluconazole resistance and increased expression of both the *ERG11* and *UPC2* genes [14]. *UPC2* is an important transcription factor that regulates the ergosterol biosynthesis pathway [52, 53]. Our results showed that isolate 50, one of the isolates resistant to posaconazole, had three mutations, D340A, E337D and Y130F, in the *ERG11* gene product, lanosterol 14α‐demethylase. A previously reported amino acid substitution in azole resistant *C. tropicalis* is Y132F, which we did not observe [54]. The isolate had a significant increase in ergosterol content, although the same was true for other isolates without missense SNPs in *ERG11*. Some studies found that the missense mutations, S258F and S113G, in *ERG3* correlate with azole resistance [29]; isolates 53, 65 and 65 have a missense mutation S112G. Interestingly, isolate 54 had a point mutation leading to the loss of the stop terminus in *ERG7*. This could have led to a larger protein product that could potentially disrupt the production of ergosterol. Isolate 54 did not significantly increase its ergosterol production but it did have a major increase in R6G output. The majority of isolates exhibited an increase in the overall output of ergosterol. These isolates likely increased ergosterol production as a means of resistance. Isolate 47, 50, 57 and 62 had mutations in the *UPC2* gene but these particular missense mutations are not known to lead to upregulation. All isolates displayed the Lys314Glu in the *ERG20* gene. Co‐occurring SNP mutations G751A and A866T in the *UPC2* gene and A395T and C461T of the *ERG11* have been found to corelate to azole‐resistance, although the mechanism requires further study [14]. None of our isolates exhibited these mutations.

The vast majority of our isolates exhibited an increase in rhodamine‐6 G efflux. The gene families, *MDR* and *CDR*, are involved; *MDR1* encodes a major superfamily (MFS) transporter and *CDR1* encodes an ATP‐binding cassette (ABC) transporter efflux pump, both of which are responsible for expelling drugs from within the yeast cells. Overexpression of these efflux pumps decreases intracellular accumulation of antifungal drugs, which is an established method of resistance in *Candida* species [19, 55]. A study that induced fluconazole resistance in *C. tropicalis* isolates demonstrated increased expression of *CDR1* and *MDR1*. Isolates that were made fluconazole susceptible expressed both genes less [8]. Clinical isolates of *C. tropicalis* , however, have significant variation in expression of *CDR1* and *MDR1* in susceptible and resistant isolates, making the relationship between overexpression of efflux‐drug pumps and resistance unclear in the species [56]. Despite having the largest output of R6G, isolates 54 and 50 had established MICs of 8 and 4 μg/mL to fluconazole, making them resistant and susceptible dose‐dependent, respectively. Compared to most other isolates, which had MICs of 16 or 64 μg/mL, the expression of efflux pumps clearly varies significantly in hospital isolates. Isolate 58, the only isolate resistant to both an azole and echinocandin, did not significantly overexpress efflux pumps. All isolates had some mutations found in *CDR1*, *CDR3* or *CDR4*, and all isolates had mutations in *CDR2*.

Our phenotypic assay of biofilm formation revealed variability in the hospital isolate's biofilm forming capabilities. In *C. tropicalis* , biofilms play central roles in both virulence and resistance to antifungals. Within the *Candida* genus, *C. tropicalis* is one of the stronger biofilm producers [42, 57, 58]. The layered structure of biofilms allows sessile cells to persist and evade the immune system. The extracellular matrix of biofilms can limit the entrance of antifungal drugs, another method of resistance. Biofilm formation by *C. tropicalis* is also linked to infections from implanted devices, such as catheters and epithelial surfaces, which could potentially lead to hospital outbreaks [19, 59]. Isolates 52, 57, 58, 62, 63 and 65 showed strong biofilm formation, on par with the control. These isolates also showed only moderate or low R6G output, indicating low efflux activity. This implies an inverse relation between biofilm formation and efflux activity; the isolates may have adopted varied methods of survival strategies in the hospital environment, with some isolates focusing on drug expulsion, while others focused on biofilm formation. However, biofilms in *Candida* species often correlate with increased efflux activity to boost drug explosion. Efflux pump genes like *CDR1*, *CDR2* and *MDR* are actually upregulated during biofilm development [60]. Interestingly, one study on *C. albicans* found that disruption of critical efflux pump genes did not affect the biofilm forming ability of mutant strains. The same study found that efflux pumps played a critical role in resistance only in early phase *C. albicans* biofilm formation [55]. This implies there is a more nuanced relationship between efflux drug pumps and their role in Candida biofilms. Biofilm formation is highly implicated in virulence [61]; only three isolates displayed significantly lower virulence in murine models compared to the control, isolates 50, 60 and 61. Of the three, isolates 50 and 61 also had lowered biofilm forming capabilities. Additionally, biofilm formation and adhesion are interlinked characteristics; adhesion is the first step in establishing biofilms on medical devices and host tissues [58]. Our results did not correlate adhesion capacity to biofilm formation, as several isolates that had lowered biofilm production did not have lowered adhesion to epithelial cells. This indicates that early attachment and downstream biofilm maturations are regulated by distinct pathways and should be investigated. Isolates 52, 57, 60, 63 and 66 displayed mutations in the *CPH2*. Isolate 46 and isolate 66 also had point mutations in the *ALS6* and *EFG1* genes respectively. These three genes are involved in numerous processes that contribute to biofilm stability [42, 62]. The variance in biofilm production and adhesion is likely due to different combinations of mutations present in each isolate.

Finally, our cell wall content showed a marked increase in the hospital isolates relative to the control strain. Chitin is an extremely important component of the *Candida* cell wall, and an increase in chitin content is a common stress response against administration of echinocandins. It has been documented that exposure to echinocandins induces upregulation of chitin synthesis in order to thicken the cell wall [43]. This leads to drug tolerance that can develop into resistance over time. Although some isolates had slightly elevated MICs to certain echinocandins, isolate 58 was the only one resistant to micafungin. Numerous isolates displayed mutations in *CHS1* and *CHS3*, two important genes related to chitin synthesis; *CHS1* is involved in the repair of damaged chitin, while *CHS3* is responsible for general chitin synthesis [63, 64]. Interestingly, one study found *Candida auris* isolates resistant to fluconazole had elevated levels of chitin that correlated with increased transcription of *CHS1* and *CHS3* [65, 66]. Mutations were found in other critical genes involved in cell‐wall construction, *FKS1* and *FKS2*. These two genes translate into the echinocandin target, β‐1,3‐glucan synthase; mutations in hot‐spot areas in these genes are known to confer resistance by changing the protein product. It should be noted that mutations in *FKS1* are tied to echinocandin resistance in *C. albicans* , while mutations in both genes are tied to resistance in *C. glabrata* [47, 67, 68]. Isolate 58 had mutations present in *CHS1*, *CHS3* and *FKS2* but did not have mutations in *FKS1*. Only two isolates had *FKS1* mutations, isolate 50 and 65, which did not display elevated MICs to any echinocandins.

To our knowledge, our study is one of the first of its kind to analyse *C. tropicalis* isolates through whole genome sequencing coupled with pathogenicity related phenotypic assays. Our data show that *C. tropicalis* isolates taken from a hospital environment displayed varied and flexible phenotypic morphologies in order to adapt to environmental pressures. Whole‐genome sequencing and phylogenetic analysis revealed isolates as highly related yet genetically structured. The isolates showed multiple closely related lineages with many shared and distinct mutations, as well as many shared and distinct phenotypes. This diversity reflects adaptive trajectories that may facilitate survival under antifungal pressure. Most of these strategies converged on increased ergosterol and chitin content, and efflux pump overexpression. Many of these phenotypic changes were associated with single nucleotide variants in genes implicated in these pathways.

## Author Contributions

**Christy Chedraoui:** data curation, formal analysis, investigation, writing – original draft. **Ibrahim Bitar:** data curation, formal analysis. **Tsolaire Sourenian:** data curation, formal analysis. **Louna El Bitar:** investigation. **Anna‐Souraya Sleiman:** validation, writing – review and editing. **Lama El Moheb:** validation, writing – review and editing. **Setrida El Hachem:** data curation, investigation. **Nour Fattouh:** methodology, validation, software, investigation. **Roy A. Khalaf:** conceptualization, methodology, funding acquisition, project administration, validation, resources, writing – review and editing. **Rola Husni:** resources.

## Funding

This work was supported by the Lebanese American University (Grant PIRF I0018), the Department of Biological Sciences, and Project Nr. Integration of Biomedical Research and Health Care in the Pilsen Metropolitan Area (Grant CZ.02.01.01/00/23_021/0008828).

## Conflicts of Interest

The authors declare no conflicts of interest.

## Supporting information


**Table S1:** Protein accession numbers of all investigated genes.

**Table S2:** Mutations identified in genes involved in drug resistance mainly.

## Data Availability

Whole‐genome sequencing data have been deposited in NCBI under BioProject accession PRJNA1454038. Additional data supporting the findings of this study are available from the corresponding author upon reasonable request.
