# Supplementary material for: Integrated Genomic and Phenotypic Analyses Reveal Convergent Resistance Patterns in Clinical Candida tropicalis Isolates
Source: Mycoses. 2026 Apr 27;69:e70181. doi: 10.1111/myc.70181 (PMC13112332; doi:10.1111/myc.70181)
Supplement: Supplementary file 1 — Table S1: Protein accession numbers of all investigated genes. [file MYC-69-e70181-s002.docx]

**Supplemental Table 1.** Protein Accession Numbers of Investigated Genes

| **Gene** | **Ascension Number** |
| --- | --- |
| *ALS1* | XP_002547996.1 |
| *ALS6* | XP_002546256.1 |
| *CDR1* | XP_002548039.1 |
| *CDR2* | KAK6867496.1 |
| *CDR3* | KAK6868659.1 |
| *CDR4* | KAK6874235.1 |
| *CHS1* | RCK65334.1 |
| *CHS3* | XP_002549288.1 |
| *CPH1* | XP_002549862.1 |
| *CPH2* | XP_002548717.1 |
| *EFG1* | AJT59418.1 |
| *ERG11* | ATO93827.1 |
| *ERG12* | KAK6885653.1 |
| *ERG2* | XP_002549356.1 |
| *ERG20* | KAK6886303.1 |
| *ERG25* | CAI5755580.1 |
| *ERG26* | XP_002549744.1 |
| *ERG3* | XP_002550182.1 |
| *ERG4* | KAK6890272.1 |
| *ERG6* | XP_002548099.1 |
| *ERG7* | RCK67838.1 |
| *ERG8* | XP_002545449.1 |
| *ERG9* | XP_002547551.1 |
| *FKS1* | XP_001526795.1 |
| *FKS2* | XP_002550363.1 |
| *HWP1* | ASK40158.1 |
| *MDR1* | KAK6881284.1 |
| *MLT1* | XP_002548912.1 |
| *MRR1* | KAK6867037.1 |
| *MSH3* | KAK6882542.1 |
| *MSH4* | KAK6890328.1 |
| *SAP3* | KAK6867641.1 |
| *SAP7* | KAK6867902.1 |
| *SAP9* | KAK6880031.1 |
| *SNQ2* | KAK6881967.1 |
| *TAC1* | XP_002551009.1 |
| *UPC2* | QEO75742.1 |
| *WOR1* | KAK6868266.1 |
