# Supplementary material for: Integrated Genomic and Phenotypic Analyses Reveal Convergent Resistance Patterns in Clinical Candida tropicalis Isolates
Source: Mycoses. 2026 Apr 27;69:e70181. doi: 10.1111/myc.70181 (PMC13112332; doi:10.1111/myc.70181)
Supplement: Supplementary file 2 — Table S2: Mutations identified in genes involved in drug resistance mainly. [file MYC-69-e70181-s001.docx]

**Supplemental Table 2.** Additional amino acid substitutions in ergosterol biosynthesis genes detected in the *C. tropicalis* hospital isolates compared to the ATCC control strain, *C. tropicalis* MYA-3404.

|  | **Isolate 46** | **Isolate 47** | **Isolate 50** | **Isolate 52** | **Isolate 53** | **Isolate 54** | **Isolate 57** |
| --- | --- | --- | --- | --- | --- | --- | --- |
| *SNQ2* | - | - | - | - | - | - | - |
| *ERG2* | - | Leu3Ser, Tyr145Asp | - | - | - | - | - |
| *ERG3* | - | - | - | - | Ser112Gly | - | - |
| *ERG4* | - | - | - | - | - | Arg6Gln | - |
| *ERG6* | - | - | - | - | - | - | Thr191Ala |
| *ERG7* | Tyr201Cys | - | His322Leu | - | - | stop_lost Ter240Trpext*? | - |
| *ERG9* | - | - | - | - | - | Ile405Val | - |
| *ERG12* | - | - | - | - | Val419Ala | Val419Ala | - |
| *ERG2*6 | - | - | - | - | - | - | - |
| *UPC2* | - | Gly392Glu | - | - | - | - | Ala251Thr, Gln289Leu |
| *FKS2* | Arg39Trp |  | Arg39Trp | - | Gly189Arg | - | - |
| *ALS6* | Val114Asp, Leu148Ile, His158Gln | - | - | - | - | - | - |
| *CPH2* | - | - | - | Lys284Asn, Lys294Arg, Lys380Glu | - | - | Glu214Lys |
| *EFG1* | - | - | - | - | - | - | - |
| *MLT1* | Glu205Lys, Glu150Asp, Asn728Asp, Tyr438Phe | - | - | Pro769Ser | - | Asn728Asp, Ser43Tyr | Asn728Asp, Ser733Pro, Pro678Leu |
| *MSH4* | - | Glu107Gly, Ala445Val | - | - | - | Ala73Val, Gln369His | Glu107Gly |
| *SAP3* | Ser9Phe, Leu14Phe, Ala29Ser, Asp33Val | Ser9Phe, Val7Leu |  | Ser9Phe, Ala29Ser, Asp33Val, Ile21Thr, Gln106His | Ser9Phe, Asn160Asp | Ser9Phe, Ala29Ser, Thr98Met | Ser9Phe, Ala29Ser, Val7Leu |
| *SAP7* | - | - | - | - | - |  | His99Gln |
| *SAP9* | - | - | - | - | Ile121Asn | Ile121Asn, Glu134Gln | - |
|  | **Isolate 58** | **Isolate 60** | **Isolate 61** | **Isolate 62** | **Isolate 63** | **Isolate 65** | **Isolate 66** |
| *SNQ2* | Arg343His | - | - | - | - | - | - |
| *ERG2* | Leu3Ser | - | - | - | - | - | Tyr145Asp |
| *ERG3* | - | - | - | - | Ser112Gly | - | Ser112Gly |
| *ERG4* | - | - | - | - | Arg6Gln | Arg6Gln | - |
| *ERG6* | - | Thr191Ala | - | - | - | - | - |
| *ERG7* | - | - | - | - | - | - | - |
| *ERG9* | - | - | Ile405Val | - | - | Ile405Val | - |
| *ERG12* | - | - | - | - | Val419Ala | - | - |
|  |  |  |  |  |  |  |  |
| *ERG2*6 | Pro282Ser, Thr144Ser | - | - | Pro282Ser | - | - | - |
| *UPC2* | - | Ala251Thr, Gln289Leu | - | Gln289Leu | - | - | - |
| *FKS2* | Gly189Arg | - | - | - | Gly189Arg | - | - |
| *ALS6* | - | - | - | - | - | - | - |
| *CPH2* | - | Glu214Lys | - | Lys284Asn, Lys294Arg, Lys380Glu | Lys380Glu | - | Lys284Asn, Lys294Arg, Lys380Glu |
| *EFG1* | - | - | - | - | - | - | Pro372Gln |
| *MLT1* | - | Pro678Leu | Ser43Tyr | Leu87Ile | - | Ser43Tyr | Ser43Tyr |
| *MSH4* | - | Gln369His | Gln369His | Glu107Gly | - | Ala73Val, Gln369His | Glu107Gly |
| *SAP3* | Ser9Phe, Ala29Ser | Ser9Phe, Ala29Ser, Val7Leu | Ser9Phe, Ala29Ser, Thr98Met | Ser9Phe, Ala29Ser, Asp33Val, Ile21Thr, Gln106His | - | Ser9Phe, Ala29Ser, Thr98Met | Ser9Phe, Ala29Ser, Asp33Val, Ile21Thr, Gln106His |
| *SAP7* | - | - | - | - | - | - | Tyr234Phe |
| *SAP9* | - | - | - | - | - | - | - |
